# Antifibrotic Therapies and Progressive Fibrosing Interstitial Lung Disease (PF-ILD): Building on INBUILD

**DOI:** 10.3390/jcm10112285

**Published:** 2021-05-25

**Authors:** John N. Shumar, Abhimanyu Chandel, Christopher S. King

**Affiliations:** 1 Department of Pulmonary and Critical Care Medicine, Walter Reed National Military Medical Center, Bethesda, MD 20814, USA; abhimanyu.chandel.mil@mail.mil; 2 Advanced Lung Disease and Transplant Program, Inova Fairfax Hospital, Falls Church, VA 22042, USA; Christopher.King@inova.org

**Keywords:** progressive fibrosing interstitial lung disease, pulmonary fibrosis, pirfenidone, nintedanib, interstitial lung disease, IPF, antifibrotics

## Abstract

Progressive fibrosing interstitial lung disease (PF-ILD) describes a phenotypic subset of interstitial lung diseases characterized by progressive, intractable lung fibrosis. PF-ILD is separate from, but has radiographic, histopathologic, and clinical similarities to idiopathic pulmonary fibrosis. Two antifibrotic medications, nintedanib and pirfenidone, have been approved for use in patients with idiopathic pulmonary fibrosis. Recently completed randomized controlled trials have demonstrated the clinical efficacy of antifibrotic therapy in patients with PF-ILD. The validation of efficacy of antifibrotic therapy in PF-ILD has changed the treatment landscape for all of the fibrotic lung diseases, providing a new treatment pathway and opening the door for combined antifibrotic and immunosuppressant drug therapy to address both the fibrotic and inflammatory components of ILD characterized by mixed pathophysiologic pathways.

## 1. Introduction

The term interstitial lung disease (ILD) describes a large, heterogeneous group of disorders affecting the lung parenchyma with overlapping clinical, radiographic, and histopathologic manifestations [1]. The underlying etiologies for ILD range broadly, including identifiable causes such as connective tissue disorders, offending drugs, and environmental triggers, as well as unclassifiable and idiopathic causes [1,2]. The most common type of idiopathic ILD is idiopathic pulmonary fibrosis (IPF). The hallmark features of IPF include progressive lung fibrosis with concomitant decline in lung function, clinical impairment, and inevitable early mortality [3]. While IPF was a disease entity initially devoid of therapeutic options, two oral antifibrotic therapies, pirfenidone and nintedanib, have demonstrated efficacy in reducing the rate of forced vital capacity (FVC) decline over one year in IPF patients with mild to moderate lung dysfunction and have been included conditionally in updated treatment guidelines for IPF [4,5,6].

**Figure 1:**
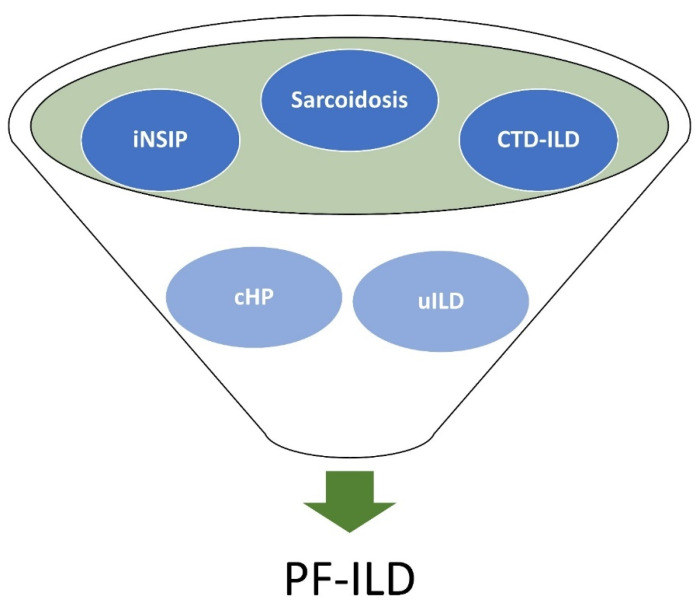
Examples of underlying etiologies in patients with PF-ILD.

Although IPF is the most common form of ILD with progressive lung fibrosis, there are other distinct types of ILD that can also share a similar progressive phenotype. Examples of these other forms of ILD with progressive lung fibrosis are displayed in Figure 1 and include idiopathic non-specific interstitial pneumonia (iNSIP), chronic hypersensitivity pneumonitis (cHP), connective tissue disease (CTD)-associated ILD, unclassifiable ILD (uILD), and sarcoidosis [1,7,8]. When associated with a progressive phenotype, these forms of ILD are broadly classified as progressive fibrosing interstitial lung disease (PF-ILD) and have significant overlap with IPF, often sharing similar radiographic, histopathologic, and clinical features [9,10,11]. The clinical outcomes for patients with PF-ILD are comparable to outcomes of patients with IPF, including the development of advanced fibrosis, progressive decline in lung function with associated dyspnea, and early mortality [7,8].

Management decisions for patients with PF-ILD have historically been challenging due to the paucity of validated therapeutic interventions. Given the phenotypic similarities to IPF and the biologically plausible benefit of the application of antifibrotic therapies to this condition, high quality evidence regarding clinical outcomes related to the administration of these medications in PF-ILD has been long awaited [7,8,12,13,14].

In this review, we provide an overview of the antifibrotic medications and their use in PF-ILD, explore ongoing studies and clinical trials, and discuss changes in the treatment paradigm for patients with PF-ILD.

## 2. Pathogenesis of Fibrosis and Genetic Variants

**Figure 2:**
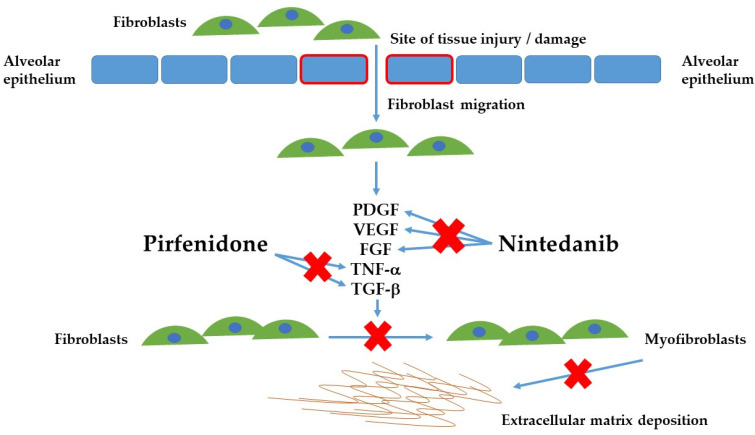
Pathophysiology of PF-ILD and the major mechanisms by which pirfenidone and nintedanib modulate steps in the cycle of lung fibrosis. Adapted from Spagnolo et al. [26]. PDGF: Platelet-derived growth factor; VEGF: Vascular endothelial growth factor; FGF: Fibroblast growth factor; TNF-α Tumor necrosis factor alpha; TGF-β: Transforming growth factor beta.

The underlying pathogenesis of PF-ILD is complex and incompletely understood. Several mechanisms have been identified in the PF-ILD phenotype, as show in Figure 2. An initial trigger of repeated inflammatory or epithelial and vascular injuries lead to cellular injury and unregulated repair [15,16,17]. Fibroblasts from the lung as well as peripheral circulation are drawn to the site of injury and are activated to myofibroblasts [17,18,19,20]. This in turn leads to secretion of an extracellular matrix resulting in stiff, dysfunctional alveolar tissue [17,18,19,20]. Further pro-fibrotic mediators are released by macrophages and lymphocytes, and in conjunction with the increase in alveolar tissue stiffness, further activate fibroblasts resulting in a self-perpetuating cycle of progressive lung fibrosis [17,18,19,20,21]. Pro-fibrotic mediators suspected to contribute to disease pathogenesis include platelet-derived growth factor (PDGF), tumor necrosis factor alpha (TNF-α), transforming growth factor beta (TGF-β), and matrix metalloproteinases [22,23,24,25]. Notably, these pro-fibrotic mediators are often elevated in patients with PF-ILD and IPF and may explain the similar clinical manifestations of these entities [22,23,24,25].

In addition to the pathogenic similarities, there have been similar genetic variants linked to both PF-ILD and IPF. The *MUC5B* rs35705950 polymorphism has been identified as a genetic risk factor for the development of IPF [25,27,28]. This genetic polymorphism has also been identified in patients with rheumatoid arthritis (RA) related ILD as well as cHP [25,27,28]. Other genetic variants include the Toll-interacting protein (*TOLLIP*) rs5743890 polymorphism and mutations in telomerase complex genes (*TERT* and *TERC*), which have been linked to survival in IPF and are seen in RA-ILD and cHP [25,29].

Acknowledging their similar pathogenesis, therapeutic intervention with antifibrotic medications such as nintedanib and pirfenidone attempts to modulate and arrest some of these steps in the cycle of lung fibrosis in patients with PF-ILD in a similar manner as IPF.

## 3. Nintedanib

Nintedanib is an oral intracellular tyrosine kinase inhibitor that targets multiple tyrosine kinases, including PDGF, vascular endothelial growth factor receptor (VEGF), and fibroblast growth factor receptor (FGFR), resulting in disruptions in the signaling pathway for fibroblast proliferation and activation [25,30]. The use of nintedanib has been widely studied in the treatment of IPF, as shown in Table 1, beginning with the Phase II TOMORROW trial in 2011 and continuing with additional Phase III data provided by the INPULSIS trials in 2014 and INSTAGE/INJOURNEY trials in 2018. The results from the TOMORROW trial suggested that nintedanib, at a dose of 150 mg orally twice daily, slowed decline in FVC, improved quality of life metrics, and decreased acute exacerbations in patients with IPF [31].

This was augmented by the data from the INPULSIS 1 and 2 trials, which showed an annual decrease in FVC −114.7 mL in the nintedanib group versus −239.9 mL with placebo for INPULSIS 1 and an annual decrease in FVC −113.6 mL in the nintedanib group versus −207.3 mL with placebo for INPULSIS 2 [4,8]. In addition to the clinical improvements demonstrated, the INPULSIS trial provided additional evidence of the safety and tolerability of nintedanib. The most significant adverse effect noted amongst patients taking nintedanib was diarrhea, which often could be managed symptomatically but did cause treatment cessation in 5–10% of patients [4,8]. Given these results, nintedanib was approved for use in IPF patients.

Extrapolated from the benefits seen in patients with IPF, the use of nintedanib was explored for patients with non-IPF fibrotic lung disease in the SENSCIS and INBUILD trials. The SENSCIS trial evaluated the efficacy and safety of nintedanib use in patients with systemic sclerosis (SSc) related ILD, some of whom were concurrently receiving immunosuppression with mycophenolate mofetil (MMF) [33]. Nintedanib was shown to reduce the annual rate of FVC decline by 44%, comparable to the previous data from the INPULSIS trials in patients with IPF, and without any treatment heterogeneity between those patients taking MMF and patients that were not [25,33]. Finally, the INBUILD trial evaluated the use of nintedanib in patients with PF-ILD [34]. INBUILD demonstrated a similar reduction in FVC decline as that documented in the previous studies. This effect was noted in patients regardless of the presence of either a usual interstitial pattern (UIP) on high resolution thoracic computed tomography (HRCT) (61% relative reduction) or non-UIP (49% relative reduction). Furthermore, the effect was consistent among varying underlying ILD diagnoses [14,25,34]. Given these results, nintedanib was approved for use in patients with SSc-ILD in 2019 and subsequently in patients with PF-ILD as well.

Building on the results of the INBUILD trial, there are multiple clinical trials addressing nintedanib use in patients with PF-ILD that are currently active. To augment the data obtained from the INBUILD trial, there is currently an active open label extension trial assessing the long-term tolerability and safety of nintedanib use in patients who completed the INBUILD trial without premature cessation of therapy (NCT03820726). Furthermore, a Japanese post-market surveillance study of patients with PF-ILD taking nintedanib is currently recruiting, with the primary objective of evaluating the incidence of adverse drug reactions (NCT04559581). Evaluating patients with pneumoconiosis-related PF-ILD, the NiPPS Trial (Nintedanib in Progressive Pneumoconiosis Study, NCT04161014) has completed patient enrollment and seeks to evaluate the annual decline in FVC in these patients on nintedanib versus placebo. In addition, the INREAL study (NCT04702893) is a planned observational study with the primary objective of investigating the correlation between changes in FVC and dyspnea or cough score as measured with the pulmonary fibrosis questionnaire over 52 weeks of nintedanib treatment in patients with PF-ILD. These results will help to guide the utilization of nintedanib in patients with PF-ILD moving forward.

## 4. Pirfenidone

Pirfenidone is an oral agent with broad anti-inflammatory, antifibrotic, and antioxidant properties. Pirfenidone primarily affects fibroblast proliferation and fibrosis-related proteins and cytokines including TGF-β and TNF-α [25,35,36,37]. The use of pirfenidone in patients with IPF has been evaluated in multiple randomized trials, as shown in Table 2, with notable studies including the CAPACITY I and II trials in 2011, the ASCEND trial in 2014, and the open-label extension RECAP trial in 2017. The CAPACITY I and II trials compared pirfenidone versus placebo in patients with IPF [38]. While CAPACITY I demonstrated improvement in FVC decline versus placebo (mean decline in FVC −8% pirfenidone versus −12.4% placebo, *p* < 0.01) and a decreased proportion of patients with a >10% decline in FVC, CAPACITY II did not demonstrate a difference in FVC decline (mean decline in FVC −9% pirfenidone versus −9.6% placebo, *p* = 0.5) [8,38].

To further elucidate the efficacy of pirfenidone in patients with IPF, the ASCEND trial randomized patients with IPF to pirfenidone or placebo [5]. The primary outcome, the number of patients with a >10% decline in FVC or death, was reduced by 47.9% in the pirfenidone group versus placebo. Furthermore, the administration of pirfenidone was associated with improved progression-free survival [5,8]. Pirfenidone subsequently received FDA approval for the treatment of IPF in 2014.

To further analyze the data regarding pirfenidone use in patients with IPF, a pooled analysis of the CAPACITY, ASCEND, and Japanese (Shionogi Phase 2 and 3 trial) data was performed by Nathan et al. [25,43]. This group noted multiple endpoints were improved with pirfenidone use including IPF-related mortality and all-cause mortality. In addition, death following one or more progression events (relative decline in percent predicted FVC >10%, absolute decline in six-minute walk distance >50 m, respiratory hospitalization) occurred less frequently in the pirfenidone intervention cohort [25,43,44].

Similar to nintedanib, the positive treatment results seen in patients with IPF treated with pirfenidone were extrapolated to those patients with PF-ILD. The LOTUSS trial first evaluated the use of pirfenidone in patients with SSc-ILD [45]. This trial assessed adverse effects in a two-week versus four-week titration group and disease outcomes. Disease outcomes remained similar to placebo and there were more treatment-emergent adverse events seen in the two-week vs. four-week titration group [25,45]. The RELIEF trial was a multi-center, double-blinded prospective trial in Germany evaluating the use of pirfenidone in a group of 127 PF-ILD patients with underlying etiologies including CTD-ILD, cHP, iNSIP, and asbestos-related fibrotic lung disease [39,40]. These patients were noted to have progression of disease even with conventional therapy, and antifibrotics were being considered due to disease progression. Patients were randomly assigned (1:1) to either pirfenidone (267 mg three times per day in week one, 534 mg three times per day in week two, and 801 mg three times per day for the remainder of the trial) versus matched placebo, added to their ongoing PF-ILD therapy. After 48 weeks, the pirfenidone group showed a significantly lower decline in FVC percent predicted compared to placebo. Although prematurely terminated due to slow recruitment, the results of this trial again suggest that in patients with PF-ILD with progressive disease despite conventional therapy, pirfenidone therapy can attenuate further decrement in FVC and disease progression [25,39,40].

There are additional trials currently ongoing regarding the use of pirfenidone in patients with PF-ILD. Multiple trials are ongoing aimed to address the efficacy and safety of pirfenidone in CTD-ILD (NCT03857854, NCT03856853). The TRAIL1 trial (NCT02808871) will evaluate the use of pirfenidone in patients with RA-ILD, with efficacy defined by progression-free survival.

## 5. A Treatment Paradigm Change

**Figure 3:**
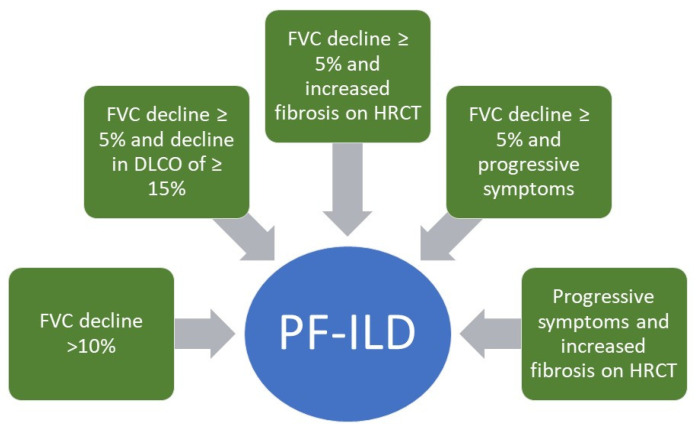
Definition of PF-ILD, adapted from George et al. [46]. Definition was developed based on inclusion criteria from available studies of antifibrotics in this condition. Progression is defined based on decrement in forced vital capacity (FVC), diffusion capacity for carbon monoxide (DLCO), progressive symptoms, or increased radiographic fibrosis as assessed by an expert thoracic radiologist, noted on high resolution thoracic computed tomography (HRCT).

PF-ILD encompasses a large, heterogeneous classification of fibrotic ILD, and management of this conglomeration of conditions continues to remain challenging. While many patients with non-IPF fibrotic lung disease show disease stabilization or improvement with immunosuppressive agents (CTD-ILD, iNSIP) and removal of antigenic stimuli (cHP), there remains a subset of this population that manifests the progressive fibrotic phenotype as defined above in Figure 3 by George et al. [46]. Nunes et al. noted that 26 of 43 patients (60%) with iNSIP, 13 of 26 (50%) patients with undifferentiated CTD-ILD, and 10 of 23 (43%) patients with defined CTD-ILD progressed despite appropriate treatment over a follow up time of 4–6 years [47]. Wijsenbeek et al. noted in a survey of ILD physicians that an estimated 18–32% of patients diagnosed with non-IPF ILD developed a progressive fibrosing phenotype and time from symptom onset to death in these patients was 61–80 months [48]. Therefore, it is of paramount importance to attempt to identify patients at risk of progressive fibrosis and initiate effective therapies to mitigate the resultant declines in FVC and diffusing capacity for carbon monoxide (DLCO), quality of life metrics, and early mortality.

The optimal management for patients with PF-ILD remains an area for further research and multidisciplinary care. The most important facets for improving outcomes in patients with PF-ILD revolve around a timely and accurate diagnosis, initiation of immunosuppressive medications at an appropriate dose with close interval follow-up testing, and initiation of antifibrotic therapies when progressive disease has been identified. The primary therapeutic intervention for patients with non-IPF ILD remains immunosuppressive agents, which can dramatically improve or stabilize the disease in some cases [49]. However, in patients with IPF, immunosuppression has been associated with harm and worse outcomes [50]. This highlights the importance of an accurate diagnosis in these patients, and tissue biopsies remain an important component of the diagnostic and treatment algorithm in appropriately selected patients [46]. If there is a decrement in lung function or worsening fibrosis on HRCT in patients with non-IPF ILD despite treatment with immunosuppressive agents, we suggest that early initiation of antifibrotic therapy should occur. Once the fibrotic cascade has started, immunosuppressant therapies alone will likely be ineffective at modulation of further clinical and radiographic decline.

Following the paradigm-shifting INBUILD trial, there are finally clinical trial results to support the biologic plausibility of antifibrotic efficacy in patients with PF-ILD with antifibrotics offering a new therapeutic option for these patients. Furthermore, the SENSCIS (nintedanib) and uILD (pirfenidone) trials demonstrated that concurrent use of both immunosuppressant medications and antifibrotics is safe and well tolerated by patients [33,42]. There is also biologic plausibility that both immunosuppression and antifibrotic therapy could address different mechanistic pathways, and thereby confer dual benefit. This has not been demonstrated in clinical trials to date, and is a much-needed area of further research to elucidate in what patients and clinical situations this could be an effective therapeutic intervention. In addition, these trials investigated the use of antifibrotic therapy as a combination treatment; the use of antifibrotics as a sequential add-on therapy has not been studied and is another area of further research [51].

Although these clinical trial results provide improved evidence to support the use of antifibrotic therapies in patients with PF-ILD, these therapies can also have drawbacks. One of the main limitations to the use of antifibrotic therapies are their associated adverse drug reactions (ADR). These have been well documented in prior studies; in the INPULSIS trials, 62.4% of patients noted diarrhea associated with nintedanib therapy, compared to 18.4% of patients receiving placebo^4^. In a study by Proesmans et al., evaluating subjective gastrointestinal side effects of patients on antifibrotic therapies, nintedanib (48.3%) users noted a significant increase in diarrhea, weight loss, vomiting, and loss of appetite compared to placebo (11.4%) [52]. Pirfenidone (40.3%) users also noted loss of appetite and weight loss [52]. Both nintedanib and pirfenidone have also been associated with elevations in liver-associated enzymes, as well [52]. Cottin et al. evaluated the long-term safety of pifenidone through the results of the PASSPORT trial, a prospective observational study of 1009 IPF patients in Europe treated with pirfenidone [53]. Seven hundred and forty-one (73.4%) patients experienced an ADR, which included nausea (208, 20.6%), fatigue (187, 18.5%), weight loss (161, 16%), and diarrhea (96, 9%); however, there were only 78 (5.5%) serious adverse drug reactions (SADR) [53]. Two hundred and ninety (28.7%) patients experienced an ADR that resulted in the discontinuation of pirfenidone; the most common ADRs that prompted discontinuation were nausea (41, 4.1%), weight loss (32, 3.2%), and rash (32, 3.2%) after a median of 99.5 days [53]. It is also important to note that 379 (51.1%) of the 741 patients who experienced an ADR reported it within 30 days of initiation of pirfenidone [53]. Factors associated with early treatment discontinuation were noted to be older age, treatment with steroids prior to pirfenidone therapy, and female sex [53].

As noted above, the adverse effects seen with nintedanib and pirfenidone can be significant, and can limit the long-term utilization of these therapies and affect quality of life metrics. As such, it is paramount to identify ways to attenuate these ADRs. Dietary interventions have been suggested, to include the use of a BRAT (bananas rice applesauce toast) diet, therapeutic intervention with antimotility or antiemetic agents such as loperamide or ondansetron, respectively, and attention to improved oral hydration [52]. Dose adjustments, through either dose reductions or interruptions in antifibrotic therapy, have also been noted to attenuate gastrointestinal adverse effects and decrease the discontinuation of therapy [52]. Cottin et al. noted that patients with ADRs related to pirfenidone were more likely to complete treatment if they had a dose adjustment (38.8%) than if they received no dose adjustment (26.1%) [53]. It appears this approach is efficacious as well. Nathan et al. noted that the annual rate of decline in FVC and the proportion of patients with IPF who experienced a decline of >50 m in 6MWD or death in a year were lower in patients who received pirfenidone at either a >90% dose intensity or <90% dose intensity compared to placebo [54]. These studies will help to guide further research and study into the extended use of these medications, and can help to guide mitigation strategies to prevent SADRs and treatment discontinuation. For example, frequent early evaluation after initiation of antifibrotic therapy can help to identify those patients with early ADRs and enable the clinician to dose adjust, offer therapeutic interventions such as antimotility or antiemetic agents, or attempt to switch to a different antifibrotic therapy.

Nintedanib and pirfenidone do not currently have generic alternatives, and as such, there can be a significant financial burden with these medications as well. Corral et al., noted a cost of $9015 per person per month for pirfenidone therapy and $10,167 per person per month for nintedanib therapy [55]. This cost burden can make antifibrotic therapies challenging to implement in lower socioeconomic settings.

Whether or not a patient would benefit from an increased dose or method of immunosuppression versus initiation of antifibrotic therapy remains a therapeutic decision which must be individualized to the patient at hand. We suggest that evidence of inflammation as demonstrated by ground glass opacities on HRCT or organizing pneumonia could lend itself to a more aggressive immunosuppressant regimen. However, if the predominant finding is pulmonary fibrosis without these additional findings, adaptation of immunosuppressant regimen is unlikely to provide additional benefit and we would argue to initiate antifibrotic therapy at that time. To address some of these uncertainties, further research must occur in developing improved tools to identify patients who are at risk for the progressive fibrosis phenotype. Biomarkers remain a promising area of research, as well as tools to identify high risk patterns on HRCT and subtle changes from baseline scans [46]. At this time, the optimal management of patients with PF-ILD remains unclear, but we argue that the results of INBUILD and other clinical trials have demonstrated the safety and efficacy of antifibrotic therapy in PF-ILD and these therapies should be utilized frequently in those patients with progressive fibrosing phenotypes.

## Figures and Tables

**Table 1:** List of major antifibrotic trials to date that have included nintedanib as an intervention. FVC: Forced vital capacity; SGRQ: St. George’s Respiratory Questionnaire; AE-IPF: Acute exacerbation of IPF.

Trial Name	Intervention	Phase and Study Design	Patient Enrollment	Primary Outcome (s)	Secondary Outcome (s)
Nintedanib					
TOMORROW [8,32] (NCT00514683, NCT01170065)	Nintedanib (50 mg daily, 50 mg twice daily, 100 mg twice daily, 150 mg twice daily) versus placebo	Phase 2, randomized, double-blind, placebo-controlled	432 patients with IPF	Annual rate of FVC decline −60 mL in nintedanib 150 mg twice daily group versus 190 mL in placebo group	Lower incidence of AE-IPF, small decrease in SGRQ with nintedanib 150 mg twice daily
INPULSIS I [4,8] (NCT01335464)	Nintedanib (Assigned in 3:2 ratio to receive either 150 mg twice daily) versus placebo	Phase 3, randomized, double-blind, placebo-controlled	515 patients with IPF	Annual rate of decline FVC −114.7 mL nintedanib versus −239.9 mL placebo (*p* < 0.01)	No significant difference in time to first AE-IPF or proportion with exacerbation
INPULSIS II [4,8] (NCT01335477)	Nintedanib (Assigned in 3:2 ratio to receive either 150 mg twice daily) versus placebo	Phase 3, randomized, double-blind, placebo-controlled	551 patients with IPF	Annual rate of decline FVC −113.6 mL nintedanib versus −207.3 mL placebo (*p* < 0.01)	Increase in time to first AE-IPF in nintedanib group and lower proportion with exacerbation in nintedanib group
SENSCIS [33] (NCT02597933)	Nintedanib (150 mg twice daily) versus placebo	Phase 3, randomized, double-blind, placebo-controlled	580 patients with SSc-ILD (48.4% who were concurrently taking mycophenolate mofetil)	Annual rate of decline FVC −52.4 mL nintedanib versus −93.3 ml placebo (*p* = 0.04).	Change from baseline in the total score on the SGRQ did not differ significantly
INBUILD [14] (NCT02999178)	Nintedanib (150 mg twice daily) versus placebo	Phase 3, randomized, double-blind, placebo-controlled	663 patients with fibrosing lung disease on HRCT	Adjusted rate of decline in the FVC was −80.8 ml per year with nintedanib and −187.8 mL per year with placebo	No significant changes in quality of life metrics as determined by the King’s Brief ILD Questionnaire

**Table 2:** List of major antifibrotic trials that have included pirfenidone as an intervention. 6MWD: Six-minute walk distance; DLCO: Diffusing capacity for carbon monoxide.

Trial Name	Intervention	Phase and Study Design	Patient Enrollment	Primary Outcome(s)	Secondary Outcome(s)
Pirfenidone					
CAPACITY I [8,38] (NCT00287716)	Pirfenidone versus placebo (randomized 2:1:2 pirfenidone 2403 mg daily, 1197 mg daily, or placebo)	Phase 3, randomized, double-blind, placebo-controlled	435 patients with IPF	Mean decline FVC −8% pirfenidone versus −12.4% placebo (*p* < 0.01)	Decreased proportion of patients with >10% decline in FVC
CAPACITY II [8,38] (NCT00287729)	Pirfenidone versus placebo (randomized 1:1 pirfenidone 2403 mg daily or placebo)	Phase 3, randomized, double-blind, placebo-controlled	344 patients with IPF	Mean decline FVC −9% pirfenidone versus −9.6% placebo (*p* = 0.5)	Reduced decline in 6MWD
ASCEND [5,8] (NCT01366209)	Pirfenidone (801 mg three times daily) versus placebo	Phase 3, randomized, double-blind, placebo-controlled	555 patients with IPF	Proportion of patients with ≥10% decline in FVC or death reduced by 47.9% pirfenidone versus placebo	Decreased decline in 6MWD, improved progression free survival
RELIEF [39,40] (EudraCT 2014-000861-32)	Pirfenidone (801 mg three times daily) versus placebo	Phase 2, randomized, double-blind, placebo-controlled	127 patients with progressive fibrosing ILD	Change in percentage of predicted FVC from baseline to week 48 by home spirometry, planned statistical model unable to be applied	Pirfenidone group less likely to have declines in FVC. Pirfenidone intervention also noted improvement in DLCO and 6MWD
INJOURNEY [8,41] (NCT02579603)	Nintedanib (150 mg twice daily) alone versus nintedanib and pirfenidone (801 mg three times daily)	Phase 4, randomized, open-label, parallel-group study	105 patients with IPF	Overall manageable safety and side effect profile (main side effect was gastrointestinal adverse effects)	Mean change in FVC were −13.3 mL and −40.9 mL in nintedanib with add-on pirfenidone vs. nintedanib alone
Maher et al. [42] (NCT03099187)	Pirfenidone (801 mg three times daily) versus placebo	Phase 2, randomized, double-blind, placebo-controlled	253 patients with progressive fibrosing ILD	Median change in FVC measured by home spirometry was −87.7 mL pirfenidone versus −157.1 mL placebo	Pirfenidone group less likely to have a decline in FVC > 5% (OR 0.42, *p* = 0.001) or >10% (OR 0.44, *p* = 0.011). Pirfenidone group also noted improvements in DLCO and 6MWD
